# Enhancing radioprotection: A chitosan-based chelating polymer is a versatile radioprotective agent for prophylactic and therapeutic interventions against radionuclide contamination

**DOI:** 10.1371/journal.pone.0292414

**Published:** 2024-04-03

**Authors:** Arthur Durand, Tatiana Borisova, François Lux, Jordyn A. Howard, Augustin Tillement, Halyna Kuznietsova, Natalia Dziubenko, Vladimir Lysenko, Laurent David, Daphné Morel, Ross Berbeco, Serhiy Komisarenko, Olivier Tillement, Eric Deutsch

**Affiliations:** 1 MexBrain, Villeurbanne, France; 2 Institute of Light and Matter, UMR 5306, University of Lyon 1-CNRS, University of Lyon 1, Villeurbanne Cedex, France; 3 Department of Neurochemistry, Palladin Institute of Biochemistry National Academy of Sciences of Ukraine, Kyiv, Ukraine; 4 Insitut Universitaire de France (IUF), Paris, France; 5 Nano-H, Fontaines Saint Martin, France; 6 Corporation Science Park, Taras Shevchenko National University of Kyiv, Kyiv, Ukraine; 7 Univ Lyon, Université Claude Bernard Lyon 1, INSA de Lyon, Université Jean Monet, CNRS, UMR 5223 Ingénierie des Matériaux Polymères (IMP), Villeurbanne Cedex, France; 8 Department of Radiotherapy, Gustave Roussy, Université Paris-Saclay, Villejuif, France; 9 Department of Radiation Oncology, Brigham and Women’s Hospital, Dana-Farber Cancer Institute, and Harvard Medical School, Boston, Massachusetts, United States of America; 10 Université Paris-Saclay, Gustave Roussy, INSERM, Radiothérapie Moléculaire et Innovation Thérapeutique, Villejuif, France; Icahn School of Medicine at Mount Sinai Department of Pharmacological Sciences, UNITED STATES

## Abstract

To mitigate the risk of radioactive isotope dissemination, the development of preventative and curative measures is of particular interest. For mass treatment, the developed solution must be easily administered, preferably orally, with effective, nontoxic decorporating properties against a wide range of radioactive isotopes. Currently, most orally administered chelation therapy products are quickly absorbed into the blood circulation, where chelation of the radioactive isotope is a race against time due to the short circulation half-life of the therapeutic. This report presents an alternative therapeutic approach by using a functionalized chitosan (chitosan@DOTAGA) with chelating properties that remains within the gastrointestinal tract and is eliminated in feces, that can protect against ingested radioactive isotopes. The polymer shows important *in vitro* chelation properties towards different metallic cations of importance, including (Cs(I), Ir(III), Th(IV), Tl(I), Sr(II), U(VI) and Co(II)), at different pH (from 1 to 7) representing the different environments in the gastrointestinal tract. An *in vivo* proof of concept is presented on a rodent model of uranium contamination following an oral administration of Chitosan@DOTAGA. The polymer partially prevents the accumulation of uranium within the kidneys (providing a protective effect) and completely prevents its uptake by the spleen.

## Introduction

In recent decades, the threat of extensive dispersion of radioactive isotopes within populated areas that would have an unfortunate effect on human health has increased drastically. These scenarios encompass the use of nuclear weapons in acts of terrorism and/or during war, as well as incidents in nuclear facilities [1]. For example, the simple detonation of a nuclear device releases over 400 radioactive isotopes, among which 40 pose significant risks to human health due to their prolonged radiological half-lives at alarming concentrations that accumulate in vulnerable organs [2]. Therefore, the development of a decorporating agent capable of effectively mitigating the effects of a wide range of isotopes is critical. Brambilla *et al*. recently compiled a comprehensive list of radionuclides that are more likely to be present in a dirty bomb, representing a diverse array of potential threats [3].

Exposure to and uptake of such contaminants can occur by various means during a nuclear incident, such as ingestion, inhalation, skin absorption, or exposure through open wounds [4]. Currently, the FDA has approved only three compounds (only one of which is used as a preventative therapy) for the treatment of exposure to specific radioactive elements, including iodine (potassium iodide, KI; approved in 1978 as a preventive therapy for the thyroid), cesium (Prussian blue; approved in 2003), and plutonium and americium (Zn-DTPA and Ca-DTPA; approved in 2004). Diethylenetriamine pentaacetate (DTPA), however, suffers from poor solubility and limited bioavailability and can only be administered intravenously (IV) or as an aerosol. Additionally, its chelation constant for U(VI) (log K = 16) is relatively low compared to many other endogenous cations [5, 6] and its rapid elimination after IV administration and toxicity concerns restrict its use as a preventive measure. IV administration also limits the use of DTPA for large-scale population treatment compared to an orally formulated medication [7]. In the case of contamination, several studies have highlighted the importance of initiating treatment as soon as possible (the urgent approach) [8], further necessitating a treatment that can be easily administered. Providing a preventative drug to a population at risk of imminent contamination, such as soldiers or workers entering a contaminated area, is also advantageous.

Recently, the use of chitosan@DOTAGA, which is composed of a chitosan backbone grafted with DOTAGA (2-[1,4,7,10-Tetraazacyclododecane-4,7,10-tris(t-butyl acetate)]-pentanedioic acid-1t-butyl ester), has been proposed as an orally administrable treatment for heavy metal contamination (lead and cadmium) in food [9, 10]. After oral administration to rodents over several days, no signs of acute or chronic toxicity were observed, and DOTAGA did not enter the blood stream and was fully eliminated from the gastrointestinal tract within 24 hours of administration [9].

The objective of the current study is to explore the potential of this polymer for use in the decorporation of a wide range of radioactive isotopes, with a specific focus on uranium. Currently, there are no suitable countermeasures available for uranium poisoning, and uranium is known to accumulate in the bones and the kidneys after entry into the bloodstream, leading to severe renal toxicity and osteosarcoma [11]. This innovative approach aims to directly chelate the radioactive cations, specifically uranium, within the gastrointestinal tract prior to their systemic absorption, which ensures their prompt elimination and mitigation of the associated toxicities.

## Results and discussion

Chitosan@DOTAGA was synthesized through a two-step process: chitosan acetylation followed by the addition of DOTAGA anhydride. The synthesis yielded in a polymer with a mass of 258 kDa, as determined by size-exclusion chromatography coupled with multiangle light scattering (SEC-MALLS). The acetylation ratio was found to be 0.29 and the grafting ratio of DOTAGA was 0.08, as determined by proton nuclear magnetic resonance (1H NMR) spectroscopy and copper titration [10].

The chelating capacity of chitosan@DOTAGA has been evaluated against a wide range of relevant metallic cations present during radioactive dispersion and in various pH environments corresponding to the gastrointestinal (GI) tract (Table 1), ranging from acidic, as within the stomach, to less acidic/neutral, as in the small intestine, colon, and rectum [12]. The experimental setup involved two aqueous solutions: a low concentration (0.01 g.L^-1^ chitosan@DOTAGA in the presence of 20 ppb each metal, mixed) and a high concentration (1 g.L^-1^ chitosan@DOTAGA in the presence of 2000 ppb each metal, mixed). Each mixture was stirred for approximately 10 hours at room temperature before undergoing ultrafiltration with a 50 kDa cutoff membrane. The metal contents within the original solution and the filtrate were then analyzed *via* ICP‒MS to determine the relative percentage of each metal that was captured and retained by the polymer.

**Table 1:** Percentages of the metals retained during ultrafiltration with two aqueous solutions: A low concentration (left, 0.01 g.L^-1^ chitosan@DOTAGA and 20 ppb metal) and high concentration (right, 1 g.L^-1^ chitosan@DOTAGA and 2000 ppb metal).

pH	Percentage of metal retained						
	Cs (I)	Ir (III)	Th (IV)	Tl (I)	Sr (II)	U (VI)*	Co (II)**
	(%)	(%)	(%)	(%)	(%)	(%)	(%)
1	5/9	6/22	14/57	8/11	6/0	5/16	5
2	5/5	5/20	57/93	4/5	4/0	5/11	8
3	3/2	6/15	89/96***	4/2	3/12	7/19	26
4	4/19	4/30	N/A	2/19	3/29	14/59	7
5	4/14	5/28	N/A	2/16	2/69	35/70	77
6	1/22	2/28	N/A	0/29	0/93	100/83***	84
7	7/51	5/54	N/A	3/56	3/95	N/A	62

Abbreviations: N/A: not applicable due to precipitation of the cation at this pH.

Notes:

* U (VI) is present in the form of UO_2_^2+^.

** Co (II) was tested only at the low concentration due to strong chelation under these conditions.

*** Partial precipitation was observed in control experiments.

Notably, strong chelation was observed for Co(II) at low concentrations, which was attributed to its high chelation constant (log K = 20.2) [13]. Significant retention was also observed for Th(IV) and U(VI), particularly at lower pH values for Th(IV) since it precipitates above pH 4. These metals showed an increase in retention capacity with increasing concentration, which is likely due to the kinetics of chelation. Interestingly, weak retention was observed at low concentrations for Ir(III), Tl(I), Cs(I), and Sr(II), even considering the high chelation constant of Sr(II) with dodecane tetraacetic acid (DOTA) (log K = 15). The retention of these metals increased significantly at higher concentrations, with Sr(II) reaching over 90% and Cs(I), Ir(III), and Tl(I) exceeding 50%, which may also be attributed to the kinetics of chelation and/or changes in the conformation of the polymer at higher concentrations, enabling easier access to chelation sites.

**Fig 1:**
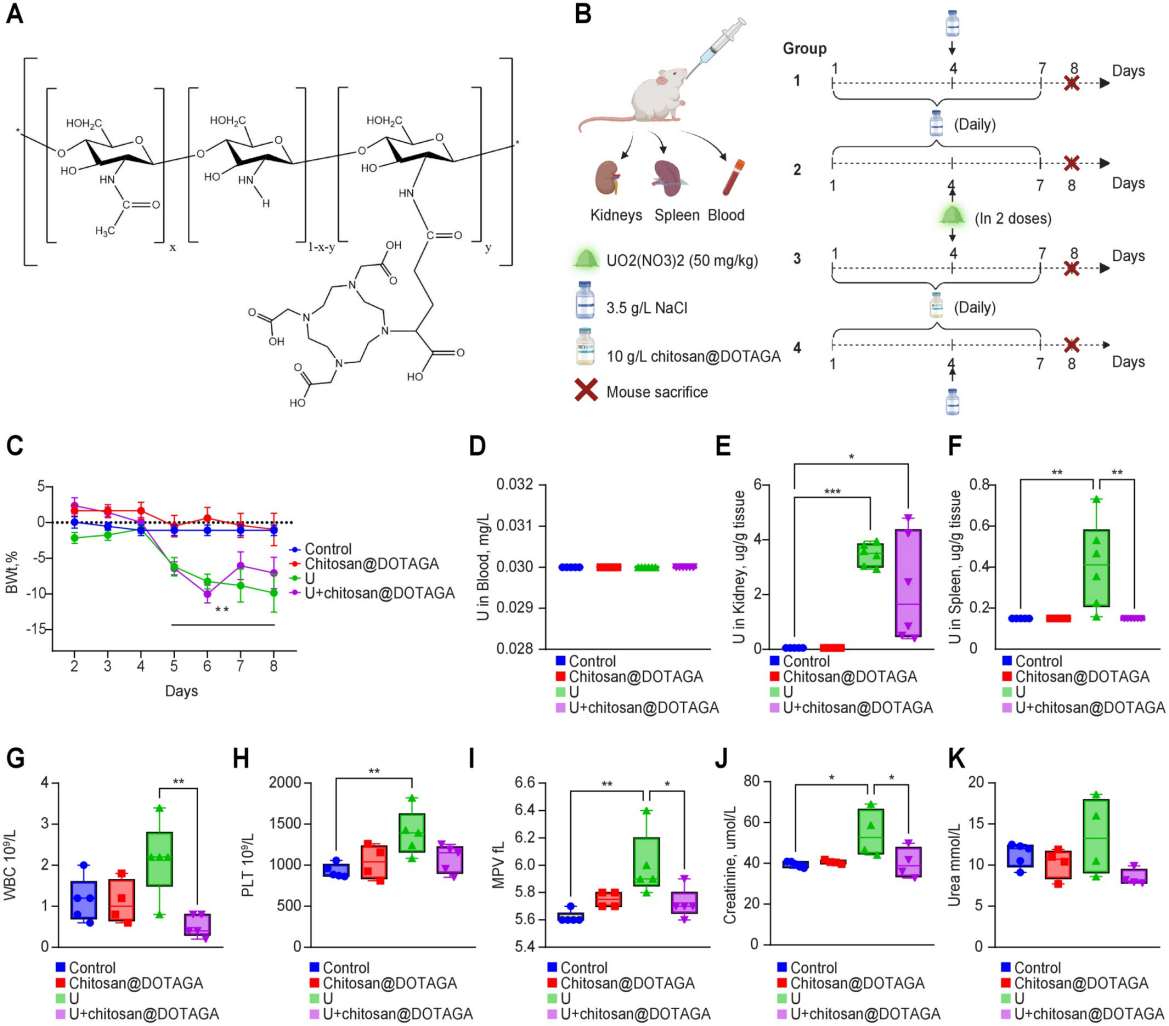
A. Representation of the structure of chitosan@DOTAGA. B. Treatment protocol for the 4 groups of mice. C. Variations in the body weights of the mice in the 4 groups. D-K. After sacrifice of the animals, the quantity of uranium was determined in the blood (D), kidneys (E), and spleen (F); the blood parameters were monitored in terms of WBC (G), PLT (H) and MPV (I) measurements; and renal function was determined by creatinine (J) and urea (K) measurements. *p<0.05, **p<0.01, ***p<0.001 compared to the respective group.

Uranium decorporation by chitosan@DOTAGA was evaluated *in vivo* in mice as a preventive and curative therapy. Four groups of mice were tested: two nonintoxicated groups administered chitosan@DOTAGA or saline orally every day for 7 days (n = 10 and 12, respectively) and oral administration of a simple saline solution on day 4; and two intoxicated groups (n = 12, each) receiving chitosan@DOTAGA or saline orally every day and saline solution containing UO_2_(NO_3_)_2_6H_2_O on day 4 (orally) to achieve a pure uranium dose of 50 mg.kg^-1^ (see Fig 1B). On day 8, all mice were sacrificed, and the blood, kidneys, and spleen were collected for uranium detection and biochemical and hematological analyses. Chitosan@DOTAGA has been administered on a daily basis thanks to its previously shown, complete elimination from the gastrointestinal tract in 24 hours with no passage in the bloodstream [9]. The proof of concept has been made with a liquid solution, but formulation will be done to propose pills for the final application.

No significant changes in body weight were observed in the two nonintoxicated groups, supporting the previously observed nontoxic nature of chitosan@DOTAGA after oral administration [9]. In the two intoxicated groups, uranium toxicity was evidenced by body weight loss, with slight recovery observed in the mice receiving chitosan@DOTAGA treatment. Four days after administration, no detectable uranium was found in the blood, which is consistent with previous literature [14]. However, uranium accumulation was observed in the kidneys and spleen, and this accumulation was partially (in the kidneys) or completely (in the spleen) prevented by chitosan@DOTAGA oral treatment. Acute uranium poisoning was evidenced by various hematological parameters, particularly elevated levels of white blood cells (WBCs) and platelets (PLTs) in the untreated group. Interestingly, no such change is observed between control groups and uranium group treated by chitosan@DOTAGA. Kidney injury was also observed due to a significant increase in serum creatinine levels and an increasing trend in urea levels in the untreated group, while a protective effect was exhibited in the mice receiving chitosan@DOTAGA treatment, who displayed normal kidney function. No indication of uranium toxicity in the liver was observed. Since the spleen is one of the main sources of platelets, spleen injury was indicated by the increases in mean platelet volume (MPV) and number of PLTs. This can be attributed to the retention of uranium in the spleen in the untreated group compared to the chitosan@DOTAGA-treated group.

These findings demonstrate that chitosan@DOTAGA can be implemented as an oral treatment both before and after radioactive contamination; moreover, chitosan@DOTAGA did not show any indications of acute toxicity as already shown in a previous study [9]. It also displays antioxidant properties that can contribute to the protection against the production of reactive oxygen species due to the presence of radioisotopes [15]. These antioxidant properties have already been shown with a conventional chitosan diet by Nishimura *et al* [16]. Interestingly, conventional chitosan has already been proposed for the decorporation of oral contamination by specific isotopes (i.e. ^60^Co and ^85^Sr) despite moderate complexation constant for Co(II) [17] and limited interaction with aqueous Sr(II) [16]. Chitosan@DOTAGA exhibits effective chelation properties across a range of pH values, targeting various metallic cations that are of significant concern in the event of radioactive dispersion. Moreover, *in vivo* proof-of-concept studies have revealed the capability of chitosan@DOTAGA to protect two vulnerable organs, the spleen and kidney, in the case of oral uranium contamination, which is notoriously challenging to prevent. The simplicity of orally administering chitosan@DOTAGA prior to and immediately following a radioactive contamination event, along with its efficacy against a diverse array of relevant radioactive cations, renders it particularly interesting for application in urgent intervention scenarios involving mass contamination, ultimately allowing the rapid protection of a population.

All *in vivo* protocols were conducted in adherence with the European Convention for the Protection of Vertebrate Animals used for Experimental and Other Scientific Purposes.

## Materials and methods

### *In vitro* ultrafiltration study

#### Polymer synthesis and characterization

The chitosan@DOTAGA polymer presented in this paper for the orally administered supplement was developed by MexBrain. Synthesis and characterization of chitosan@DOTAGA were performed as described by Natuzzi *et al*. and Howard *et al*. in previous studies [9, 10]. The chitosan was purchased from Matexcel (Bohemia, NY, USA, reference number NAT-0030), the DOTAGA anhydride was purchased from CheMatech (Dijon, France), the 1,2-propanediol and acetic anhydride were purchased from Sigma Aldrich (Saint-Quentin-Fallavier, France), and the acetic acid was purchased from VWR (France). ^1^H NMR spectra were performed at the NMR Platform of Institut de Chimie de Lyon (Axel’One Campus-Lyon) on a Bruker Avance III spectrometer 400.1 MHz and analyzed using Top Spin software. Samples were dissolved in D2O at 5 g.L^−1^. The NMR characterization evidences that 28% of the chitosan monomers are acetylated, and in conjunction with the HPLC—UV data, 7% of the monomers house a molecule of grafted DOTAGA while 65% of monomers remain de-acetylated in the form of Glucosamine (GlcN) which is in accordance with previous results [9, 10]. The weight (*M*_*w*_) and number (*M*_*n*_) average molar masses of chitosan@DOTAGA were determined by size exclusion chromatography coupled with refractive index and multi-angle laser light scattering measurements and calculated using the method previously described by Schatz *et al*. (see S1 File) [18].This formulation is soluble in water ranging from pH 2–9 with and without salts and provides a stability up to 50 g.L^-1^.

#### Ultrafiltration

For the ultrafiltration experimentation, 20 mL Sartorius Vivaspin^®^ centrifugation tubes with an internal membrane of 50 kDa were used. The centrifugation was performed at 4000 rpm for 10–30 minute cycles at a time. 20 mL of the solution were passed through the membrane, until the volume above the membrane reached about 1 mL. After the centrifugation, the solution that had passed through the membrane was collected for each sample, henceforth referred to as the undernatant as well as the solution remaining above the membrane, henceforth referred to as the supernatant.

#### ICP-MS analysis

The original solutions were prepared using metal standards purchased from SCP Science with concentrations of 1000 ppm of Co, Th, Tl, Cs, Sr, or U in 5% HNO3 or 1000 ppm of Ir in 10% HCl, a chitosan@DOTAGA solution and water. Two concentration domains were chosen, with the same ratio in concentrations between chitosan@DOTAGA and metals for both: the first one with 0.01 g.L^-1^ chitosan DOTAGA and 20 ppb of each metal, and the second one 1 g.L^-1^ chitosan@DOTAGA and 2000 ppb of each metal (without cobalt, this one showing a strong chelation even at the low concentrations domain). The pH was adjusted using 1 M NaOH and 1 M HCl. For each pH and concentration domain, two solutions were created: one solution with chitosan@DOTAGA and metals, and another solution with only metals as a control of the experiment.

The samples, including the original solution, the supernatant, and the undernatant, were analyzed by ICP-MS to determine the concentration of metals of interest within each solution as follows. For the low concentration domain, the samples were diluted by a factor of 100 for the undernatants and the original solutions and of 1000 for the supernatants using 1% HNO3. For the high concentration domain, the samples were diluted by a factor of 10^4^ for the undernatants and the original solutions and of 10^5^ for the supernatants using 1% HNO3. An internal standard (indium) was added to each sample with a final concentration of 2 ppb In. The analyses were performed in standard mode using a Perkin Elmer NexION2000 mass spectrometer equipped with Syngistix software (Licensed software by Perkin Elmer. Version: 2.3 (Build 2.3.7916.0)). A calibration curve ranging from 0.001 to 0.2 ppb for Co, Cs, Ir, Th, Tl, Sr and U was created to allow the system to convert the counts per second recorded for each sample into a concentration in ppb. After analysis, a threshold of detection of 0.01 ppb was applied to the concentrations obtained.

For each metal, the percentage of metal captured by the polymer *%*_*captured*_ has been calculated using the metal mass in the undernatants *m*_*UN*_ and the metal mass in the original solutions *m*_*ON*_ as described by the following formula:

$${\%}_{captured}=1-\frac{m_{UN}}{m_{i}}$$

### *In vivo* study of uranium toxicity mitigation by chitosan@DOTAGA

All experiments with the animals were conducted in compliance with bioethics principles, legislative norms and provisions of the European Convention for the Protection of Vertebrate Animals used for Experimental and Other Scientific Purposes (Strasbourg, 1986), General Ethical Principles for Experiments on Animals, adopted by the First National Bioethics Congress (Kyiv, 2001), and ARRIVE and Animal Care guidelines. Protocol of the study was approved by Palladin Institute of Biochemistry Animal Care and Use Committee (Protocol # 3 dated March 28, 2023).

#### Mice

This study design, animal selection, handling, and treatment were all in accordance with ARRIVE and Animal Care guidelines (Palladin Institute of Biochemistry of the NASU Animal Care and Use Committee, general protocol for experiments using animals #1 dated Jan 10 2022). All mice are drug and test naïve. The mouse strain C57Bl6 was used for this study. This strain of mice is commonly used for toxicity and efficiency studies, and therefore is an appropriate choice for this study. Animals had free access to standard or enriched rodent chew and free access to boiled tap water. A seven-day acclimation period was respected before the study began. Group randomization was based on body weight. All *in vivo* protocols are in adherence with the European Convention for the Protection of Vertebrate Animals used for Experimental and other Scientific Purposes. On the 8th day of the study (24h after the last administration) mice were anesthetized by 2,2,2-tribromoethanol (150 mg/kg) (Sigma-Aldrich, USA) and sacrificed by cervical dislocation. During the study animals were observed for visual clinical signs daily according to the FELASA recommendations [19].

Clinical signs to define humane endpoints included loss of body weight of 20%, dehydration, recumbency, lethargy, respiratory distress, inability to reach food and water, and persistent hypothermia.

#### Chemicals and equipment

Uranyl nitrate hexahydrate (UO_2_(NO_3_)_2_*6H_2_O, CAS 13520-83-7) was purchased from Sigma-Aldrichhttp://en.chembase.cn/company-15.html. Hematology analyzer MCL-3124 (MeCan Medical, China), Fully Auto Chemistry Analyzer MF-240 (MedFuture LLC, USA), and a Shimadzu ICPE 9820 (Shimadzu Corporation, Tokyo, Japan) were employed as analytical tools within this study for hematology, biochemistry, and spectrometry, respectively.

#### Analytical procedures

The activity of AST, ALT, ALP, GGT, LDH enzymes (liver functional activity markers), creatinine and urea content (kidney functional activity markers) and total protein (TP) content were assessed in blood serum, using commercial kits according to the manufacturer’s instructions (Cormay). Uranium was detected in whole blood, kidney and spleen tissues by inductively-coupled plasma optical atomic emission spectrometry method after samples microwave mineralization in concentrated nitric acid solution with added H_2_O_2_. Hematology analysis was performed by Hematology Analyzer MCL-3124 (MeCan Medical, China).

#### Dose levels, group division, and sampling

Mice were gavaged daily with a 10 g.L^-1^ chitosan@DOTAGA solution in the volume 5 mL.kg^-1^ to achieve 50 mg.kg^-1^ dose of chitosan@DOTAGA for 7 days. On the 4th day they were given orally with the saline solution of uranium salt UO_2_(NO_3_)_2_*6H_2_O in the volume 5 mL.kg^-1^ to achieve the dose of 50 mg.kg^-1^ of pure uranium. The overall dose of uranium was split on 2 administrations 4 h in between.

In all, the study included four groups (See Fig 1B):

Mice were gavaged daily with either a 10 g.L^-1^ chitosan@DOTAGA solution in the volume 5 mL.kg^-1^ to achieve a dose of 50 mg.kg^-1^ of chitosan@DOTAGA for 7 days, or a 3.5 g.L^-1^ saline solution (0.2 mL per day). On the 4^th^ day, an additional solution of either a saline solution of uranium salt UO_2_(NO_3_)_2_*6H_2_O in the volume 5 mL.kg^-1^ to achieve the dose of 50 mg.kg^-1^ of pure uranium (the overall dose of uranium was split on 2 administrations 4 h in between), or a 3.5 g.L^-1^ saline solution was provided in addition to the daily dose of chitosan@DOTAGA.

Hematology and biochemical (AST, ALT, ALP, GGT, LDH, creatinine, urea and TP) analyses, and blood and tissue analyses for uranium were performed on the 8th day of following the sacrifice.

#### Group characteristics

C57Bl/6 female mice were 13 weeks old, the initial body weight ranged from 16 to 21 g. Average body weight across all experimental groups was 18.5 g (SD = 1.0 g, CV = 5.7%). Body weight was measured every day between 8–9 AM.

#### Frequency and duration of the treatment

Animals were treated with chitosan@DOTAGA or a saline solution for 7 days. On the 4^th^ day animals were additionally treated with uranium salt solution. chitosan@DOTAGA and saline treatments were orally administered once per day between 8–9 AM (10 mL.kg^−1^). Uranium treatment was orally administered twice with 4 h between the doses. The first dose was given 1 h after chitosan@DOTAGA/saline treatment.

#### Statistical analysis

Before performing statistical analysis, the normality and homoscedasticity of the samples were assessed using the Shapiro test. Mixed-effects analysis or one-way ANOVA analysis with Tukey post-hoc test were applied to assess the statistical significance between groups. All analyses were performed using GraphPad Prism V.9.0.0 software.

## Supporting information

S1 File (DOCX)
