# Supplementary material for: Enhancing radioprotection: A chitosan-based chelating polymer is a versatile radioprotective agent for prophylactic and therapeutic interventions against radionuclide contamination
Source: PLoS One. 2024 Apr 3;19(4):e0292414. doi: 10.1371/journal.pone.0292414 (PMC10990188; doi:10.1371/journal.pone.0292414)
Supplement: S1 File — (DOCX) [file pone.0292414.s001.docx]

**Supplementary Information:**

Enhancing radioprotection: A chitosan-based chelating polymer is a versatile radioprotective agent for prophylactic and therapeutic interventions against radionuclide contamination

Arthur Durand,^1,2^ Tatiana Borisova,^3^ François Lux,^2,4,*^ Jordyn A. Howard,^1,2^ Augustin Tillement,^2,5^ Halyna Kuznietsova,^6^ Natalia Dziubenko,^6^ Vladimir Lysenko,^2^ Laurent David,^7^ Daphné Morel,^8^ Ross Berbeco,^9^ Serhiy Komisarenko,^3^ Olivier Tillement,^2^ Eric Deutsch^10,*^

^1^MexBrain, 13 Avenue Albert Einstein, 69100 Villeurbanne, France

^2^Institute of Light and Matter, UMR 5306, University of Lyon 1-CNRS, University of Lyon 1, Villeurbanne Cedex, France

^3^Department of Neurochemistry, Palladin Institute of Biochemistry National Academy of Sciences of Ukraine, 9 Leontovicha Street, Kyiv 01054, Ukraine

^4^Insitut Universitaire de France (IUF), Paris, France

^5^Nano-H, 305 rue des fours 69270 Fontaines Saint Martin, France

^6^Corporation Science Park, Taras Shevchenko National University of Kyiv, 60 Volodymyrska Str., Kyiv 01033, Ukraine

^7^Univ Lyon, Université Claude Bernard Lyon 1, INSA de Lyon, Université Jean Monet, CNRS, UMR 5223 Ingénierie des Matériaux Polymères (IMP), 15 Bd A. Latarjet, 69622 Villeurbanne Cedex, France

^8^Université Paris-Saclay, Gustave Roussy, Department of Radiotherapy, 94805, Villejuif, France

^9^Department of Radiation Oncology, Brigham and Women’s Hospital, Dana-Farber Cancer Institute, and Harvard Medical School, Boston 02115, USA

^10^Université Paris-Saclay, Gustave Roussy, INSERM, Radiothérapie Moléculaire et Innovation Thérapeutique, 94800, Villejuif, France

* Corresponding authors: François Lux and Eric Deutsch

**Materials and methods**

*Determination of polymer weight and number average molar masses*

The weight (*M_w_*) and number (*M_n_*) average molar masses of chitosan@DOTAGA were determined by size exclusion chromatography coupled with refractive index and multi-angle laser light scattering measurements. Size determination studies were carried out with a VWR degasser, an Agilent 1260 Infinity pump whose flow rate was fixed at 0.5 mL.min^-1^, an Agilent 1260 Infinity ALS automatic injector (injected volume: 100 µL), an Agilent 1260 Infinity TCC oven (30°C), a detector UV Agilent 1260 Infinity MWD VL (λ = 280 nm), a Wyatt DAWN HELEOS II MALLS detector (laser λ = 664 nm, Fused Silica cell), a Wyatt Optilab Trexrefractometer with λ = 658 nm (25°C), 2 columns: TSK PW G6000 and TSK PW G2500 (Tosoh). The analysis was carried out with a 0.2 mol.L^-1^ acetate buffer eluent (pH = 4.8), an analysis time of 60 minutes and samples at a concentration of 1 mg mL^-1^. To perform the SEC analysis, the samples were first filtered through a 0.45 μm CME filter. The *dn/dc* ratio was determined on samples whose concentration ranges from 0.1 mg.mL^-1^ to 1 mg.mL^-1^ prepared using the buffer solution previously filtered on a 0.1 µm CME filter. Mw was calculated using the obtained *dn/dc* ratio and the following equations (Schatz *et al.*, *Biomacromolecules*, **2003**;**4**(3):641-8):

$$\frac{K^{'}c}{R(\theta, c)}=\frac{1}{M_{w}P(\theta)}+2A_{z}c$$

$$P\left( \theta\right)\approx1-\frac{16\pi^{2}n_{0}^{2}R_{g}^{2}}{3\lambda^{2}}{sin}^{2}\left( \frac{\theta}{2} \right)+o({sin}^{4}\left( \frac{\theta}{2} \right))$$

Where:

- *R* is an experimental coefficient dependent on the intensity of the light scattered at a certain angle (*θ*) and concentration (*c*).
- *Mw* is the mass average molar mass.
- *A_z_* is the second virial coefficient in the virial expansion of the osmotic pressure
- *K’* is a constant defined as

$$K^{'}=\frac{4\pi^{2}{(\frac{dn}{dc})}^{2}n_{0}^{2}}{N_{a}\lambda^{4}}+2A_{z}c$$

- *N_a_* is the Avogadro’s number
- *n_0_* is the refractive index of the solvent
- *λ* is the laser wavelength
- *P(θ)* is a function describing the angular dependence of the scattered light, related to the gyration ratio *R_g_*

**Table S1. Control experiment at low concentration of retention of metals using ultrafiltration depending of pH.** Percentage of retained metals during ultrafiltration of the metal-only solutions at low metal concentration (20 ppb)

|  | Percentage of metal retained (20 ppb metals only) | | | | | | |
| --- | --- | --- | --- | --- | --- | --- | --- |
| pH | Cs (I) | Ir (III) | Th (IV) | Tl (I) | Sr (II) | U (VI)* | Co (II) |
| 1 | 10% | 10% | 16% | 9% | 6% | 10% | 7% |
| 2 | -1% | -1% | 10% | 0% | -4% | 2% | 1% |
| 3 | -1% | -3% | 41% | -2% | -1% | 2% | -4% |
| 4 | 3% | 2% | N/A | -2% | 2% | 12% | 4% |
| 5 | 1% | 1% | N/A | -2% | -4% | 13% | -1% |
| 6 | 2% | 1% | N/A | 1% | 3% | 67% | -3% |
| 7 | -1% | 3% | N/A | 0% | 1% | N/A | 6% |

*N/A: Not applicable due to precipitation of the cation at this pH.*U(VI) is present under UO_2_^2+^ form.*

**Table S2. Control experiment at high concentration of the retention of metals using ultrafiltration depending of pH.** Percentage of metal retained during ultrafiltration of the metal-only solutions at high metal concentration (2000 ppb)

|  | Percentage of metal retained (2000 ppb metals only) | | | | | |
| --- | --- | --- | --- | --- | --- | --- |
| pH | Cs (I) | Ir (III) | Th (IV) | Tl (I) | Sr (II) | U (VI)* |
| 1 | 2% | 5% | 6% | 2% | 2% | 3% |
| 5 | 4% | 7% | N/A | 2% | 0% | 4% |

*N/A: Not applicable due to precipitation of the cation at this pH.*U(VI) is present under UO_2_^2+^ form.*


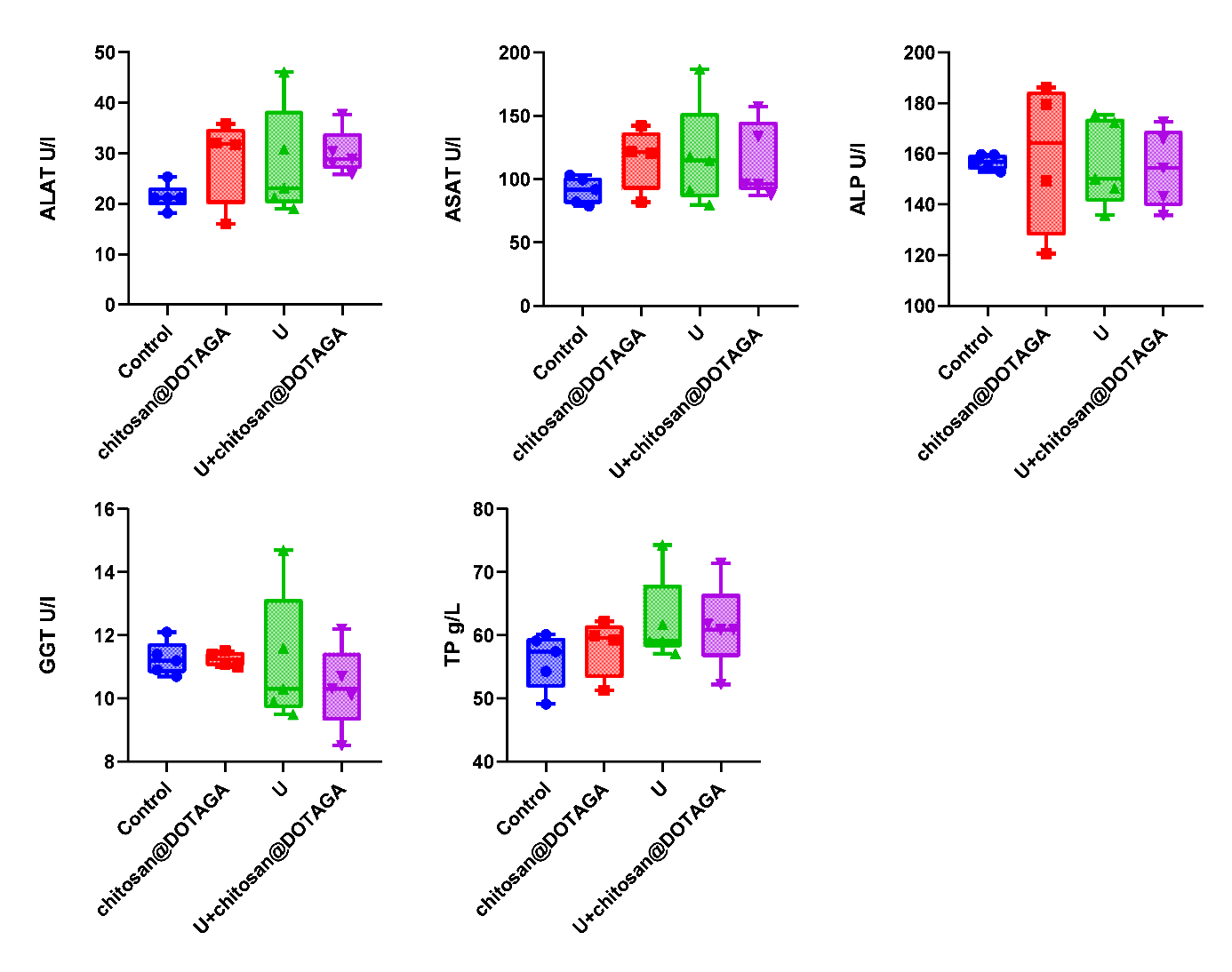


**A**

**B**

**C**

**D**

**E**

**Fig. S1. Liver enzyme profile after per os administration of different formulations.** A-D After sacrifice, the activities of ALT (A), AST (B), ALP (C), and GGT (D) enzymes were measured, as well as total protein (TP) content (E).

**
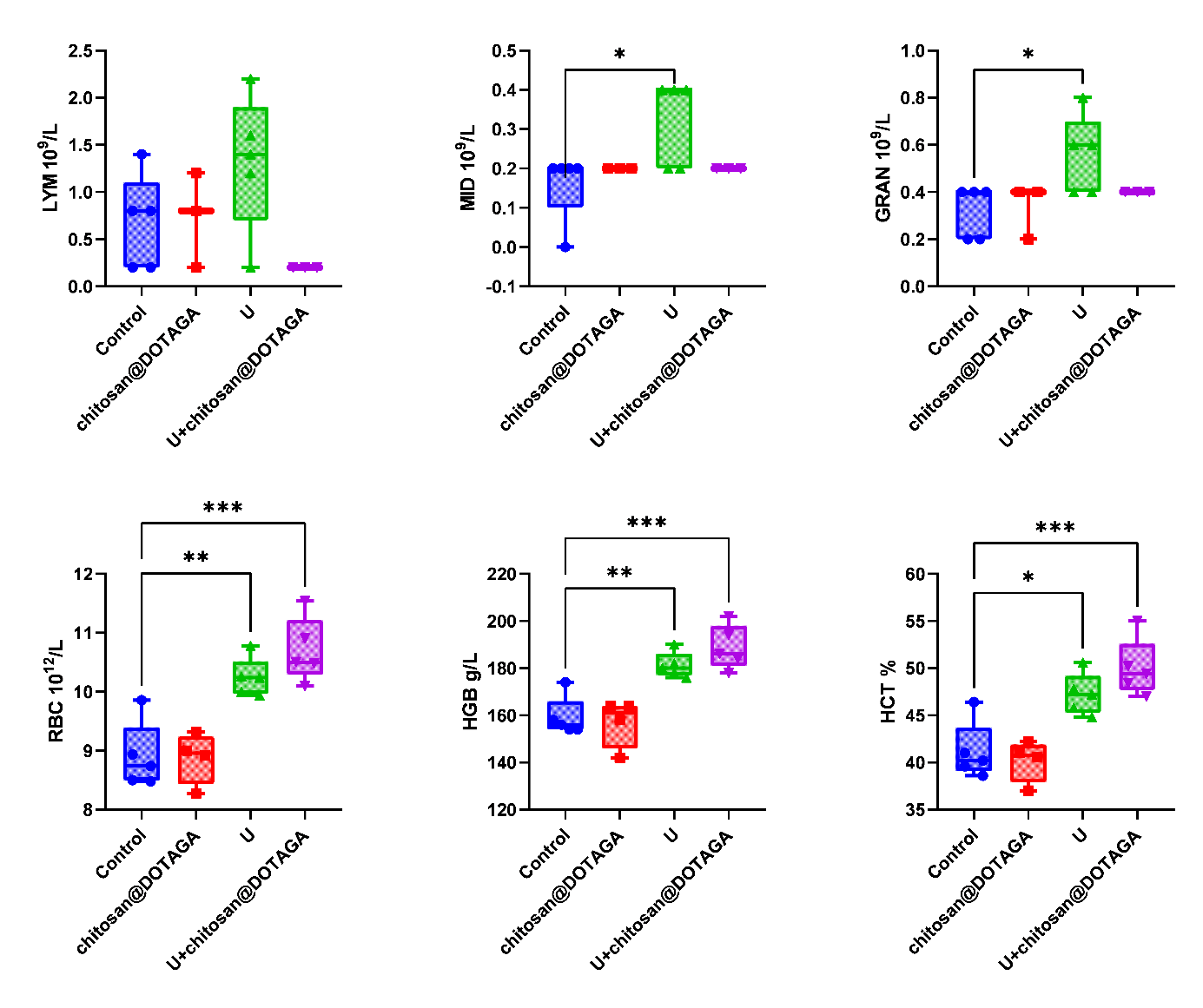
**

**A**

**B**

**C**

**D**

**E**

**F**

**Fig. S2. Hematological parameters after per os administration of different formulations.** After sacrifice, lymphocytes (LYM) (A), monocytes and eosinophils (MID) (B), granulocytes (GRAN) (C), red blood cells (RBC) (D), hemoglobin (HGB) (E) amounts and hematocrit (HCT) (F) percentage were measured.


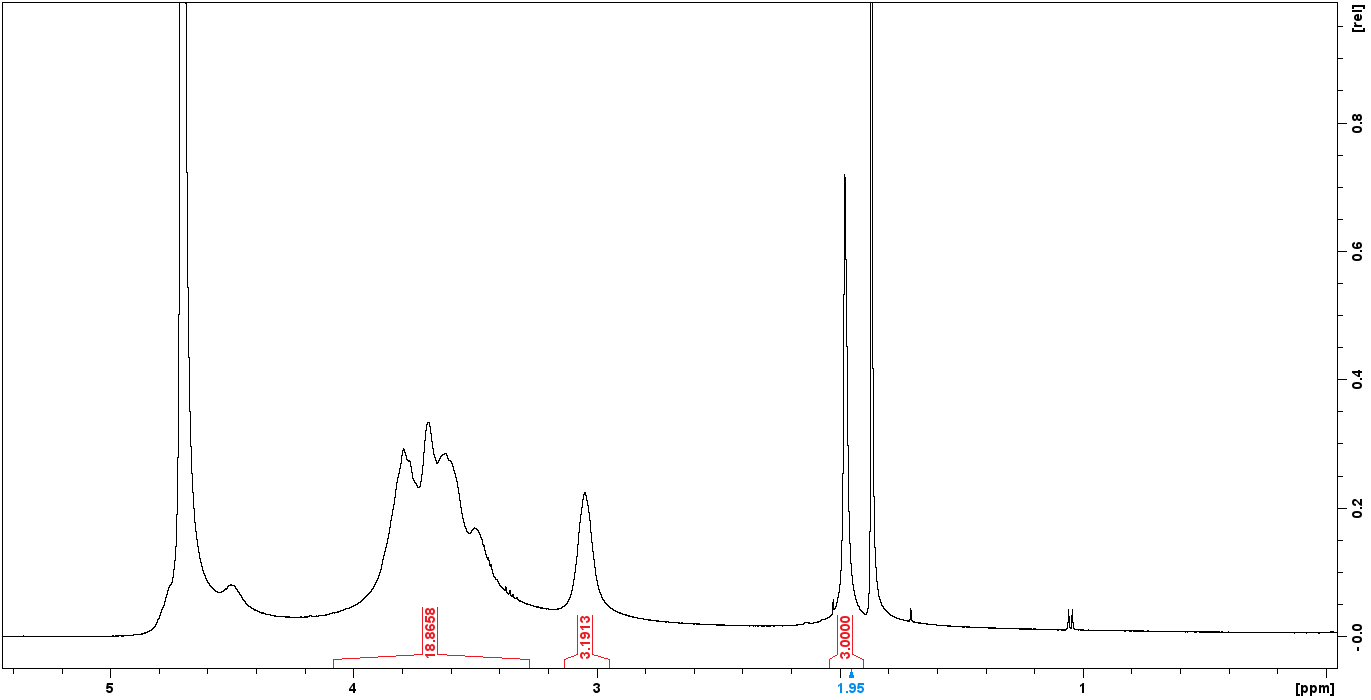


**Fig. S3. ^1^H NMR spectrum of reacetylated chitosan in D_2_O at 5 g.L^-1^.** Resonances from 2.9 ppm to 4.1 ppm corresponds to 6 protons of the glucosamine subunits, peak at 2 ppm corresponds to the 3 protons of the acetyl group of the N-acetyl-D-glucosamine subunit. Solvent peak at 1.9 ppm is attributed to residual acetate group from acetic acid and doublet at 1.1 ppm corresponds to residual 1,2-propanediol. Acetylation degree is calculated using the Hirai method (Hirai *et al.*, *Polym. Bull.*, 1991).


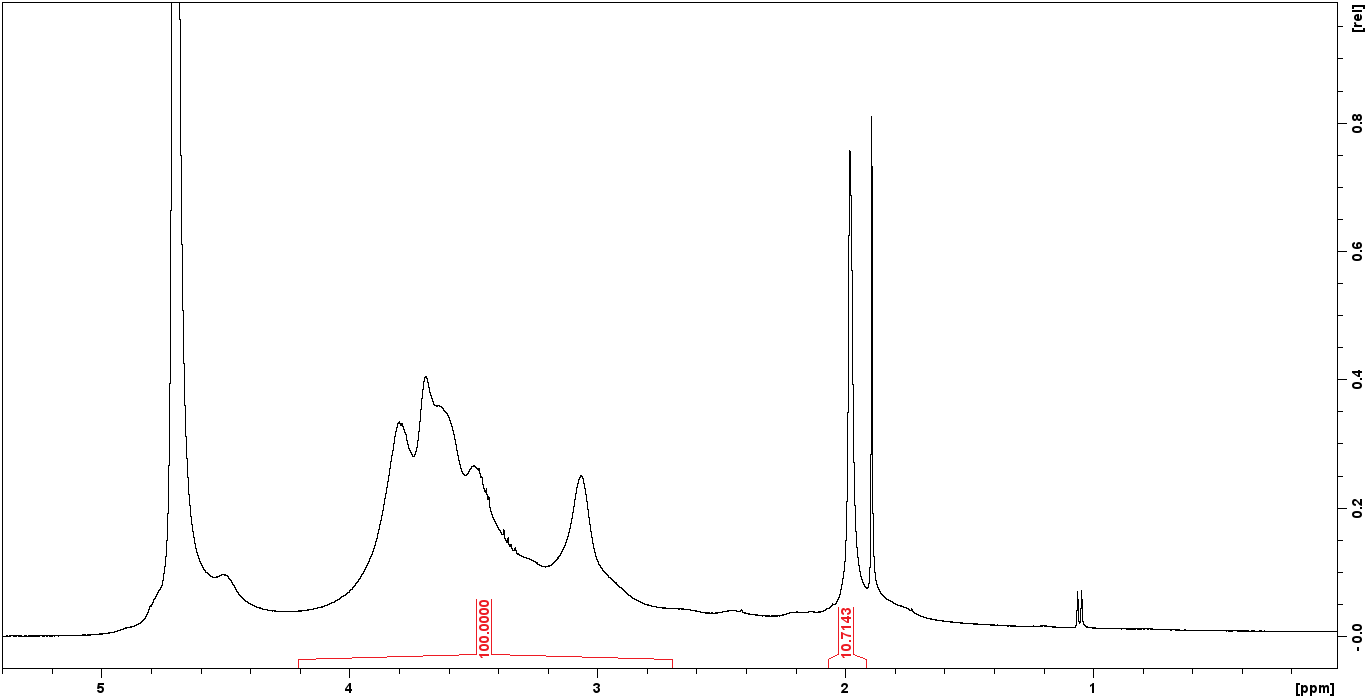


**Fig. S4. ^1^H NMR spectrum of chitosan@DOTAGA in D_2_O at 5 g.L^-1^.** Resonances from 2.9 ppm to 4.1 ppm corresponds to 6 protons of the glucosamine subunits and 25 protons of the DOTAGA moiety, peak at 2 ppm corresponds to the 3 protons of the acetyl group of the N-acetyl-D-glucosamine subunit. Solvent peak at 1.9 ppm is attributed to residual acetate group from acetic acid and doublet at 1.1 ppm corresponds to residual 1,2-propanediol. ^1^H NMR spectrum is similar to previous study (Natuzzi *et al.*, *Sci. Rep.*, 2021).
